# Association between Health Literacy and Radiation Anxiety among Residents after a Nuclear Accident: Comparison between Evacuated and Non-Evacuated Areas

**DOI:** 10.3390/ijerph15071463

**Published:** 2018-07-11

**Authors:** Yujiro Kuroda, Hajime Iwasa, Masatsugu Orui, Nobuaki Moriyama, Chihiro Nakayama, Seiji Yasumura

**Affiliations:** 1 Department of Public Health, Fukushima Medical University, Fukushima Prefecture 960-1295, Japan; hajimei@fmu.ac.jp (H.I.); oruima@fmu.ac.jp (M.O.); moriyama@fmu.ac.jp (N.M.); nakac@fmu.ac.jp (C.N.); yasumura@fmu.ac.jp (S.Y.); 2 Tokyo Metropolitan Institute of Gerontology, Tokyo 173-0015, Japan

**Keywords:** health literacy, radiation anxiety, nuclear accident, risk perception

## Abstract

Following the accident at the Fukushima Nuclear Power Plant in March 2011, both experts and the national government provided insufficient information on radiation, leading to widespread distrust in the community. This study aimed at clarifying the association between anxiety about radiation and health literacy among residents in evacuation and non-evacuation areas in Fukushima. A questionnaire survey was sent to randomly sampled residents between August and October 2016, and data from 777 responses (38.9% valid response) were analyzed. The questionnaire assessed current radiation anxiety and discrimination and prejudice based on radiation exposure through seven items and communicative and critical health literacy through five items. Multiple regression analysis of the association between radiation anxiety and health literacy showed that the level of health literacy was significantly negatively associated with radiation anxiety in the evacuation areas (marginally in the non-evacuation areas) and marginally negatively associated with discrimination and prejudice in the evacuation areas but not in the non-evacuation areas. Therefore, improving health literacy could alleviate radiation anxiety.

## 1. Background

After the March 2011 disaster at the Fukushima Daiichi Nuclear Power Plant, anxiety about radiation grew among both the evacuees and non-evacuees in Fukushima. Some people continue to have mental and physical problems attributable to radiation anxiety [1]. The literature on previous nuclear accidents, such as those at Three Mile Island and Chernobyl, suggests that the most substantial effects of nuclear accidents on local residents were long-term mental health problems [2,3]. One study suggested that radiation anxiety increased perceptions of health-related risks [4]. After the Fukushima accident, mental health problems increased among the evacuees [5,6]. These mental health problems are a long-term effect. Radiation effects can be categorized as acute (hair loss, skin symptoms, nosebleeds, etc.), delayed (for example, cancer occurrence), and genetic (expressed in descendants). Suzuki et al. (2015) examined residents’ mental health on the Kessler Psychological Distress Scale (K6) and their risk perception, asking what residents thought about the chances that each type of radiation effect would occur; they found a high proportion of people with mental health problems among those who thought that radiation effects were very likely [7]. Thus, there was a relationship between the high rate of mental health effects and increased health-related risk perception.

Health literacy can be understood as the perception of health-related risks. According to Nutbeam’s analysis of the concept, there are two distinct schools of thoughts: one views health literacy as a clinical risk, and the other understands it as a personal asset [8]. While the risk model focuses on an individual’s degree or ‘prior status derived from existing literacy and numeracy’, the asset model focuses on skill or ‘an outcome to health patient education’. Although the former is largely limited to clinical applications or used in clinical settings, the latter can be applied to community settings [8]. The concept of health literacy in the asset model is represented by the World Health Organization (WHO) definition: ‘the cognitive and social skills which determine the motivation and ability of individuals to gain access to, understand and use information in ways which promote and maintain good health’ [9,10]. A review of the literature on different concepts of health literacy will be certainly valuable for further development of health literacy studies; however, it is beyond the scope of this study. We chose the asset model for the purposes of this study for the following two reasons: The first reason is a technical one; to measure people’s health literacy, we used the measurement model, communicative and critical health literacy, developed by Ishikawa et al. [11]. This model reflects the WHO definition of communicative and critical health literacy [11]. By following the WHO definition, we wanted to maintain the integrity of our study. The second reason is to understand and examine the degree of people’s health literacy in a very specific situation after the Fukushima nuclear accident, where risk communication failed to reach a public consensus on radiation effects. There was little, and extremely limited, literacy and numeracy available regarding radiation effects. In such a setting, the asset model can better describe the construction of people’s health literacy.

Furthermore, it should be noted that an association between people’s health literacy and well-being has been pointed out by previous studies. Low health literacy is associated with poorer understanding of diseases, misunderstanding prescriptions and increased medication errors, poorer understanding of nutrition labels, and decreased use of disease prevention services (health check-ups, vaccinations, and the like) [12,13,14]. It can lead to frequent use of emergency medical services, high hospitalization rates, poor management of chronic diseases (diabetes, hypertension, asthma, HIV/AIDS, and so on), bad self-rated health status, and even high mortality rates [12]. For people with poor health literacy, it is difficult to gain new knowledge, understand what they are advised, have confidence in what they do, and change their behaviors [13]. In contrast, health-related knowledge can be improved by giving out easy-to-understand printed materials, and providing verbal instructions can lead to [14] healthy behaviors [15].

The risk communication after the Fukushima nuclear accident was conducted poorly. The government (and experts) failed to provide residents with proper evacuation orders, which caused them to receive unnecessary radiation exposure in the early stage of the nuclear disaster. Considering the insufficient distribution of radiation-related information provided to residents after the Fukushima nuclear accident, we suggest that the collapse of community trust in experts and the government became the biggest obstacle to the uptake of measures to counter the fallout. Yamashita (2014) argued that the Fukushima event was not only a health disaster but also an information disaster [16]. Other studies have pointed out the difficulty of building effective communication between information providers (experts and the government) and recipients (community members) in the absence of trust [17,18,19]. Experts and the government failed to earn the trust of residents in Fukushima and other areas. But, they kept sending out one-sided information, further contributing to the divide. In our previous survey, 72.2% of the respondents agreed that they could not determine which advice—out of so much—was right [20]. In a situation in which information is abundant, residents in affected areas must develop the skills to recognize the right information: they need to improve their health literacy.

In this study, we aimed at clarifying the association between anxiety about radiation and health literacy among residents in evacuation and non-evacuation areas in Fukushima. We suggest a basis for guidelines for interpersonal communication by aid workers, such as public health nurses and counselors, in disaster response through activities to improve health literacy among residents in affected areas.

## 2. Methods

### 2.1. Target Population

This survey targeted 2000 residents of Fukushima Prefecture aged 20–79. We divided Fukushima Prefecture into four areas based on the general regional classification of Aizu, Nakadōri, Hamadōri, and the evacuation area (the restricted area, evacuation prepared area, and deliberate evacuation area as determined on 22 April 2011) and selected 500 people from each area. The selection was based on a two-stage stratified random sampling (stage one, survey of region, stage two, of individuals). A total of 33–34 individuals per point were randomly selected from municipal resident registration files to obtain 2000 representative participants. Nakadōri and Hamadōri included local municipalities that were partially in the evacuation area; these were included in the evacuation area.

The survey used in the present study, entitled “Survey of Health and Information”, was administered as an anonymous, self-reporting postal questionnaire. The survey period was between 15 August and 17 October 2016. In total, 916 people responded. After incomplete or inadequate answers from 139 people were excluded, we analyzed the remaining 777 responses (38.9%). The present study was approved by the Fukushima Medical University’s Ethics Committee (approval number: 2699). We considered a returned questionnaire as participant consent to the objective of the study and their voluntary participation in it.

### 2.2. Study Items

#### 2.2.1. Main Outcome

Radiation anxiety was defined as the degree of negative cognition and perception, such as worries and anxieties, about the possible adverse health effects of radiation exposure and related psychosocial problems—specifically, perceived stigma and discrimination due to radiation exposure [21]. The items assessed were selected from a qualitative analysis of descriptions of worry, anxiety, and problems related to radiation exposure by the evacuees in Fukushima Prefecture [22] (see Table 1).

The respondents were asked to answer, “strongly agree” (4 points), “agree” (3), “disagree” (2), or “strongly disagree” (1). A person with radiation anxiety will score 4–16 points, and a person with discrimination and prejudice will score 3–12 points [1]. In a previous study, these items had a coefficient of reliability by internal consistency (Cronbach’s α) of 0.812, and the Pearson’s coefficient of the correlation between the radiation anxiety score and both depressive and somatic symptoms was 0.28–0.35 [21].

#### 2.2.2. Key Items

We assessed the respondents on their communicative and critical health literacy [11], rating their ability to (i) collect health-related information from various sources, (ii) extract the information sought, (iii) understand and communicate the obtained information, (iv) consider the credibility of the information, and (v) make decisions on the basis of the information. Each goal was rated on a 5-point scale ranging from 1 (never) to 5 (strongly agree), with a median of 3 and the average of the five items as the score. The respondents were categorized into two groups: a high health literacy (HL) group (above the median) and a low-HL group (below the median). According to Ishikawa et al., the internal consistency of the five items was adequately high (Cronbach α = 0.86). The item-total correlations were all positive and ranged from 0.77 to 0.85 [11].

#### 2.2.3. Relevant Factors

Information on the respondents included sex, age, educational background, family arrangement, employment status, exercise habits, sleep quality, drinking habits, smoking habits, and self-rated health status. All the information was binarized (see Table 2).

### 2.3. Analysis

First, we calculated the frequency and proportions of the basic characteristics and relevant factors in both areas and then calculated χ^2^ test statistics for differences between the areas. Second, we calculated the frequency and proportions of the low- and high-HL groups and then calculated the χ^2^ test statistics for the differences between the groups. Third, we calculated the average score and standard deviation on the radiation anxiety and discrimination and prejudice subscales for each basic characteristic and relevant factor and then used a two-sample *t*-test to test the differences. Finally, to examine the associations between health literacy and radiation anxiety in each area, we used the scores of both subscales as the outcome in each area and then analyzed these outcomes by multiple regression analysis models that incorporated health literacy and relevant factors.

We considered using psychometric approaches that target general populations to analyze the scores on the radiation anxiety scale, but as the characteristics of the population affected by the Fukushima nuclear accident were different from those of the general population, we conducted a factor analysis instead. To examine the factor structure of the radiation anxiety scales, we performed a confirmatory factor analysis using the robust maximum likelihood estimation, as the data deviated from normal. We assessed a one-factor model and a two-factor model; the two-factor model included the radiation anxiety (items 1–4) and the discrimination and prejudice (items 5–7) subscales.

We calculated two indexes of model fit: the root-mean-square error of approximation (RMSEA) and the comparative fit index (CFI). Model fit is deemed “adequate” when RMSEA ≤ 0.08 and CFI ≥ 0.90 and “excellent” when RMSEA ≤ 0.06 and CFI ≥ 0.95 [23,24].

## 3. Results

### 3.1. Respondent Characteristics

The average age of the respondents was 55.4 ± 14.7 years (range, 20–79 years). Women constituted 55% of the total (Table 2). By area, 186 respondents (23.9%) came from Hamadōri, 189 (24.3%) from Nakadōri, 231 (29.7%) from Aizu (606 or 78.0% from non-evacuation areas), and 171 (22.0%) from evacuation areas. The employment rate was significantly greater in the evacuation areas (57.4%) than in the non-evacuation areas (33.4%, *p* < 0.001). However, the proportion of good self-rated health was lower in the evacuation areas (35.1%) than in the non-evacuation areas (49.6%, *p* < 0.001; Table 2).

### 3.2. Results of Factor Analysis of Radiation Anxiety

The one-factor model had few acceptable model fit values (χ^2^ = 158.6, df = 14, *p* < 0.01, RMSEA = 0.115, CFI = 0.912). In contrast, the two-factor model had adequate fit values (χ^2^ = 60.2, df = 13, *p* < 0.01, RMSEA = 0.068, CFI = 0.971). Thus, we used the two-factor model. Cronbach’s α was 0.844 for all seven items, 0.837 for items 1–4 (factor 1) and 0.695 for items 5–7 (factor 2). The correlation between factors 1 and 2 was 0.582 (*p* < 0.01).

### 3.3. Health Literacy and Radiation Anxiety by Region

There was no significant difference in the average scores of health literacy between areas. The score for radiation anxiety was significantly higher in the evacuation areas (10.2 ± 3.0) than in the non-evacuation areas (9.5 ± 2.9, *p* = 0.006). The score for discrimination and prejudice based on radiation exposure was also significantly higher in the evacuation areas (7.3 ± 2.1) than in the non-evacuation areas (5.9 ± 2.0, *p* < 0.001).

### 3.4. Association between Characteristics of Respondents, Health Literacy, and Radiation Anxiety

The respondents were allocated into two groups by the median of their health literacy scores: the high-HL group included 451 people (58.0%) and the low-HL group included 326 people (42.0%; Table 3). The groups differed significantly in educational background, exercise habits, and self-rated health status. The high-HL group had more tertiary (51.8%) than secondary graduates (36.5%, *p* < 0.001), more people who exercised (48.2%) than did not (34.7%, *p* < 0.001), and more people with good self-rated health status (47.6%) than bad (37.1%, *p* = 0.003). On the radiation anxiety subscale, the respondents with employment scored higher (10.1 ± 2.8) than those without employment (9.4 ± 2.9, *p* = 0.001), the respondents with unsatisfactory sleep quality scored higher (9.9 ± 2.8) than those with satisfactory sleeping quality (9.1 ± 3.0, *p* < 0.001), and the respondents with bad self-rated health status scored higher (10.4 ± 2.8) than those with good self-rated health status (8.9 ± 2.8, *p* < 0.001). On the discrimination and prejudice subscale, the respondents with unsatisfactory sleeping quality scored higher (6.5 ± 2.2) than those with satisfactory sleeping quality (5.6 ± 1.9, *p* < 0.001), the respondents who smoked scored higher (6.5 ± 2.2) than those who did not (6.1 ± 2.1, *p* = 0.041), and the respondents with bad self-rated health status scored higher (6.6 ± 2.2) than those with good self-rated health status (5.8 ± 1.9, *p* < 0.001).

### 3.5. Association between Health Literacy and Radiation Anxiety or Discrimination and Prejudice by Region

Radiation anxiety was associated with self-rated health status in both areas. It was significantly negatively related to health literacy in the evacuation area (*p* = 0.028) and marginally negatively associated with healthy literacy in the non-evacuation area (*p* = 0.052; Table 4). It was also significantly negatively associated with being employed in the non-evacuation area. Moreover, discrimination and prejudice based on radiation exposure was significantly negatively associated with sleep quality and significantly positively associated with self-rated health status in both areas. It was also significantly negatively associated with age in the evacuation area. There was no association with health literacy in the non-evacuation area (*p* = 0.754), but there was a marginal negative association in the evacuation area (*p* = 0.078). The model accuracy (adjusted *R*^2^) on the radiation anxiety subscale was 0.105 in the evacuation area and 0.071 in the non-evacuation area. That on the discrimination and prejudice subscale was 0.150 in the evacuation area and 0.043 in the non-evacuation area.

## 4. Discussion

We examined radiation anxiety among residents in Fukushima by comparing their characteristics between the evacuation and non-evacuation areas, and we examined the association between radiation anxiety and health literacy of people randomly selected from the general population. Low health literacy was associated with increased radiation anxiety—significantly in the evacuation area and marginally in the non-evacuation area.

The residents of Fukushima came to distrust scientific information. Previous studies suggested that their distrust stemmed from information overload and polarization of information in the media [25]. The conventional approach to risk communication involves explaining the health effects of radiation directly to the public [19]. But, the results of this study suggest a new approach to improving health literacy: that is, providing access to the appropriate scientific information and, more importantly, increasing the public’s capacity to recognize the appropriate information from the vast amount available. This approach could be effective in reducing radiation anxiety.

Discrimination and prejudice based on radiation exposure differed between areas: the association with health literacy was marginally significant in the evacuation area but not in the non-evacuation area. Although some residents from the evacuation area remained in Fukushima, some evacuated to other prefectures (and even other countries). The latter group experienced discrimination, feeling it necessary to hide knowledge of their hometown from others [26]. Moreover, because everyone had a different view on health effects of radiation, they also felt it necessary to hide their own view to avoid potential disputes with others. Our results show that the evacuees had an increased chance of developing the same type of stigma.

More importantly, this study suggests a possibility of reducing radiation anxiety by improving health literacy among the populace in evacuation areas likely to be stigmatized. The discrimination and prejudice that the evacuees faced was not due to the poor health literacy of the residents themselves but could be due to the poor health literacy of their new neighbors who, through a misunderstanding of radiation effects, could display discrimination and prejudice. Having high health literacy could allow the evacuees to respond rationally to such treatment.

Various risk communication activities have been carried out by the government, doctors, and researchers to reduce radiation anxiety among residents. Examples include dialogue with the public as part of the Fukushima Health Management Survey [19], Yorozu health counseling at municipal health check-ups [19], communication on health promotion between experts and residents in Iitate and Kawauchi villages [25,27,28], and discussions during whole-body counter examination [29,30]. All these activities were initiated as group-based discussions but then gradually shifted to bidirectional communication between experts and residents on comprehensive health risks and individual well-being. Our results newly suggest that it is possible to reduce radiation anxiety by fostering people’s capacity to choose the appropriate health information—that is, to improve their health literacy—and that this approach could be more effective than previous communication activities in evacuation areas.

In addition to poor health literacy, unemployment in the non-evacuation area was associated with radiation anxiety. Even after adjustment for other variables, being unemployed still raised radiation anxiety. This suggests that the quality of information differs according to how much time residents spend at home and how much they interact with others outside the home—for example, at work. There was no significant negative association of discrimination and prejudice based on radiation exposure with health literacy. Nevertheless, radiation anxiety and concerns about discrimination were greater among the non-elderly people in the evacuation area. This result suggests that the non-elderly people could develop self-stigmas more easily because many of them were evacuated to distant places where the chance to face discrimination could be higher [31].

Following the accident, initiatives were begun to improve the health literacy of public health nurses and nursery teachers. A maternal and child health support group analyzed the results of 18-month-age check-ups and records of discussions between mothers and community nurses in Fukushima City [32]. The results showed that the mothers’ confidence in childcare was associated with their interpersonal relationships at home; moreover, a high proportion of mothers who disagreed with other family members over risk perceptions of radiation effects showed depressive tendencies. Community nurses are often the first health-care professionals with whom residents come into contact, most often providing health services in the form of check-ups, counseling, and education. Despite their crucial role in the community, community nurses in Fukushima were neither prepared to address community needs nor provided with tools to help them communicate as needed. Being aware of the difficulty in transferring scientific knowledge and information to residents, the nurses insisted on the need to improve their communication skills so as to provide mothers with the health-related information that they need. To meet this need, health literacy training has been offered to public health nurses in Fukushima since 2013 [33].

## 5. Strengths and Limitations

Although the random selection of participants is a strength of this study, there are some limitations. First, being a cross-sectional study, it cannot establish causation between health literacy and health anxiety about radiation. Second, despite the help of the local government, the number of responses was limited; some of the evacuees were still displaced and were thus potentially excluded from the survey. Third, certain important variables related to anxiety, such as socioeconomic status, were not evaluated in our study. Fourth, and last, the study cannot reveal, or even suggest, the mechanism by which health literacy influences anxiety about radiation: to do so requires further in-depth qualitative studies. In particular, we will need to examine how radiation anxiety develops in people with low anxiety and how it is reduced in people with high anxiety. We will also need to examine their information-seeking behaviors—to be precise, how they tried to obtain health-related information—after the nuclear accident.

## 6. Conclusions

Low health literacy could prevent individuals from obtaining appropriate information in the course of a nuclear disaster. Subsequently, the lack of appropriate information could increase their health anxiety about radiation. These results show that improving the health literacy of the general population of Fukushima Prefecture could help in preventing anxiety about radiation.

Moreover, we found a significant association between poor health literacy and discrimination and prejudice based on radiation exposure among respondents in the evacuation areas but not in the non-evacuation areas. Though speculation, considering the fact that evacuees are more likely to face discrimination and prejudice due to their new neighbors’ misunderstanding of radiation effects, this result raises the possibility that improving the health literacy not only of evacuees but also of the general public could be a means of lowering the degree of discrimination and prejudice.

## Figures and Tables

**Table 1:** Construction of a radiation anxiety scale [1,21,22].

Subscale	Questions
Radiation anxiety	(i) I am concerned about getting a serious illness in the future due to the effects of radiation. (ii) Every time I feel ill, I am afraid this is caused by radiation exposure. (iii) I am concerned that radiation effects can be inherited by the next generation, such as children and grandchildren. (iv) I feel strong anxiety when I see news reports concerning the nuclear power plant accident.
Discrimination and prejudice based on radiation exposure	(v) I have had the experience of being discriminated against (or unfairly treated) because I lived in an area that is reported to have high levels of radiation. (vi) I try not to tell others that I am a resident of that area as far as possible. (vii) I have experienced conflicts and trouble with my family members over radiation health effects.

**Table 2:** Characteristics of research subjects.

Basic Characteristics	Area		*p* Value
	Non-Evacuation Area (*n* = 606)	Evacuation Area (*n* = 171)	
Age			
Non-elderly	406 (67.0)	109 (64.1)	0.52
Elderly	200 (33.0)	61 (35.9)	
Sex			
Male	279 (46.0)	75 (43.9)	0.664
Female	327 (54.0)	96 (56.1)	
Education			
Secondary	378 (62.7)	120 (71.0)	0.046
Tertiary	225 (37.3)	49 (29.0)	
Family arrangement			
Single household	65 (10.7)	26 (15.3)	0.107
Other	540 (89.3)	144 (84.7)	
Work			
Employed	201 (33.4)	97 (57.4)	<0.001
Other	401 (66.6)	72 (42.6)	
Exercise			
Yes	284 (47.3)	76 (44.4)	0.543
No	316 (52.7)	95 (55.6)	
Sleep quality			
Satisfactory	194 (32.2)	44 (25.7)	0.111
Unsatisfactory	408 (67.8)	127 (74.3)	
Drinking			
Yes	174 (29.0)	54 (32.0)	0.447
No	427 (71.0)	115 (68.0)	
Smoking			
Yes	121 (20.1)	39 (23.1)	0.393
No	480 (79.9)	130 (76.9)	
Self-rated health status			
Good	299 (49.6)	60 (35.1)	0.001
Bad	304 (50.4)	111 (64.9)	

Note: By age group, the respondents were divided into “non-elderly” (20–64) and “elderly” (65 years and older). By educational background, the respondents were divided into “secondary” (middle school and high school graduates) and “tertiary” (junior college, vocational school, and university graduates). By family arrangement, they were divided into “single household” and “other” (couple; couple with unmarried offspring; single parent with unmarried offspring; or three-generation extended family). The other divisions were “employed” (working) versus “other” (seeking work or not working); “exercise 1–3 times a week or more (Yes)” versus “no exercise (no)”; “satisfactory sleep” versus “unsatisfactory sleep” (slightly dissatisfied, quite dissatisfied, very dissatisfied, or not sleeping at all); “drink alcohol (yes)” versus “don’t drink (no)”; “smoke (yes)” versus “don’t smoke (no)”; and self-rated health status “good” (extremely good, very good, or good) versus “bad” (fair or unhealthy).

**Table 3:** Relationship between basic characteristics, health literacy, and radiation anxiety.

Basic Characteristics	Health Literacy (HL)			Score on Radiation Anxiety Scale	*p* Value	Score on Discrimination and Prejudice Based on Radiation Exposure Scale	*p* Value
	Low HL Group (*n* = 451)	High HL Group (*n* = 326)	*p* Value				
Age							
Non-elderly	296 (57.5)	219 (42.5)	0.701	9.6 ± 2.9	0.439	6.3 ± 2.2	0.024
Elderly	154 (59.0)	107 (41.0)		9.8 ± 2.9		6.0 ± 2.1	
Sex							
Male	211 (59.6)	143 (40.4)	0.423	9.5 ± 3.0	0.165	6.3 ± 2.2	0.379
Female	240 (56.7)	183 (43.3)		9.8 ± 2.8		6.2 ± 2.1	
Education							
Secondary	316 (63.5)	182 (36.5)	<0.001	9.7 ± 3.0	0.615	6.1 ± 2.2	0.133
Tertiary	132 (48.2)	142 (51.8)		9.6 ± 2.7		6.3 ± 2.0	
Family arrangement							
Single household	54 (59.3)	37 (40.7)	0.822	9.4 ± 3.3	0.408	6.1 ± 2.3	0.548
Other	396 (57.9)	288 (42.1)		9.7 ± 2.8		6.2 ± 2.1	
Work							
Employed	268 (56.7)	205 (43.3)	0.369	10.1 ± 2.8	0.001	6.3 ± 2.2	0.214
Other	179 (60.1)	119 (39.9)		9.4 ± 2.9		6.1 ± 2.1	
Exercise							
Yes	213 (51.8)	198 (48.2)	<0.001	9.7 ± 2.9	0.923	6.2 ± 2.2	0.871
No	235 (65.3)	125 (34.7)		9.7 ± 2.9		6.2 ± 2.1	
Sleep quality							
Satisfactory	130 (54.6)	108 (45.4)	0.236	9.1 ± 3.0	<0.001	5.6 ± 1.9	<0.001
Unsatisfactory	318 (59.4)	217 (40.6)		9.9 ± 2.8		6.5 ± 2.2	
Drinking							
Yes	125 (54.8)	103 (45.2)	0.264	9.4 ± 2.7	0.072	6.3 ± 2.2	0.303
No	321 (59.2)	221 (40.8)		9.8 ± 2.9		6.2 ± 2.1	
Smoking							
Yes	96 (60.0)	64 (40.0)	0.59	9.7 ± 2.9	0.81	6.5 ± 2.2	0.041
No	351 (57.5)	259 (42.5)		9.7 ± 2.9		6.1 ± 2.1	
Self-rated health status							
Good	188 (52.4)	171 (47.6)	0.003	8.9 ± 2.8	<0.001	5.8 ± 1.9	<0.001
Bad	261 (62.9)	154 (37.1)		10.4 ± 2.8		6.6 ± 2.2	

**Table 4:** Radiation anxiety and stigma-related factors in each area.

Related Factors	Radiation Anxiety				Discrimination and Prejudice Based on Radiation Exposure			
	Evacuation Area		Non-Evacuation Area		Evacuation Area		Non-Evacuation Area	
	β	*p*	β	*p*	β	*p*	β	*p*
Sex	−0.065	0.463	−0.010	0.820	−0.148	0.090	−0.027	0.543
Age	−0.006	0.951	−0.033	0.460	−0.257	0.004	−0.048	0.289
Education	−0.032	0.699	0.032	0.451	0.051	0.524	0.076	0.078
Family arrangement	0.009	0.909	0.065	0.104	0.012	0.878	0.072	0.077
Work	0.000	0.997	−0.127	0.004	0.020	0.809	−0.070	0.117
Exercise habits	0.125	0.119	0.020	0.633	0.067	0.393	0.006	0.881
Sleep quality	−0.045	0.570	−0.075	0.079	−0.171	0.026	−0.138	0.001
Drinking habits	0.057	0.497	−0.079	0.073	0.106	0.195	0.005	0.918
Smoking habits	−0.106	0.210	0.039	0.356	−0.103	0.211	0.078	0.073
Self-rated health status	0.329	<0.001	0.187	<0.001	0.248	0.002	0.108	0.015
Health literacy	−0.176	0.028	−0.080	0.052	−0.137	0.078	−0.013	0.754
Model accuracy (adjusted *R*^2^)	0.105		0.071		0.150		0.043	
*N*	164		590		164		590	

Note: β indicate regression coefficient; the level of significance at *p* < 0.05.
